# A comprehensive trial on PFAS remediation: hemp phytoextraction and PFAS degradation in harvested plants†

**DOI:** 10.1039/d3va00340j

**Published:** 2024-01-05

**Authors:** Sara L. Nason, Sara Thomas, Chelli Stanley, Richard Silliboy, Maggie Blumenthal, Weilan Zhang, Yanna Liang, Jasmine P. Jones, Nubia Zuverza-Mena, Jason C. White, Christy L. Haynes, Vasilis Vasiliou, Michael P. Timko, Bryan W. Berger

**Affiliations:** a The Connecticut Agricultural Experiment Station New Haven CT 06511 USA sara.nason@ct.gov; b Upland Grassroots Limestone ME 04750 USA; c Mi'kmaq Nation Presque Isle ME 04679 USA; d Department of Environmental and Sustainable Engineering, University at Albany, State University of New York Albany NY 12222 USA; e University of Minnesota Minneapolis MN 55455 USA; f Yale School of Public Health, Department of Environmental Health Sciences New Haven CT 06510 USA; g University of Virginia, Department of Biology Charlottesville VA 22903 USA; h University of Virginia, Department of Chemical Engineering Charlottesville VA 22903 USA

## Abstract

Per- and polyfluoroalkyl substances (PFAS) are a class of recalcitrant, highly toxic contaminants, with limited remediation options. Phytoremediation – removal of contaminants using plants – is an inexpensive, community-friendly strategy for reducing PFAS concentrations and exposures. This project is a collaboration between the Mi'kmaq Nation, Upland Grassroots, and researchers at several institutions who conducted phytoremediation field trials using hemp to remove PFAS from soil at the former Loring Air Force base, which has now been returned to the Mi'kmaq Nation. PFAS were analyzed in paired hemp and soil samples using targeted and non-targeted analytical approaches. Additionally, we used hydrothermal liquefaction (HTL) to degrade PFAS in the harvested hemp tissue. We identified 28 PFAS in soil and found hemp uptake of 10 of these PFAS. Consistent with previous studies, hemp exhibited greater bioconcentration for carboxylic acids compared to sulfonic acids, and for shorter-chain compounds compared to longer-chain. In total, approximately 1.4 mg of PFAS was removed from the soil *via* uptake into hemp stems and leaves, with an approximate maximum of 2% PFAS removed from soil in the most successful area. Degradation of PFAS by HTL was nearly 100% for carboxylic acids, but a portion of sulfonic acids remained. HTL also decreased precursor PFAS and extractable organic fluorine. In conclusion, while hemp phytoremediation does not currently offer a comprehensive solution for PFAS-contaminated soil, this project has effectively reduced PFAS levels at the Loring site and underscores the importance of involving community members in research aimed at remediating their lands.

## Abstract 2

In this community driven project, hemp plants were used to extract PFAS from contaminated soil and hydrothermal liquefaction was used to degrade PFAS in the harvested hemp.

## Graphical abstract

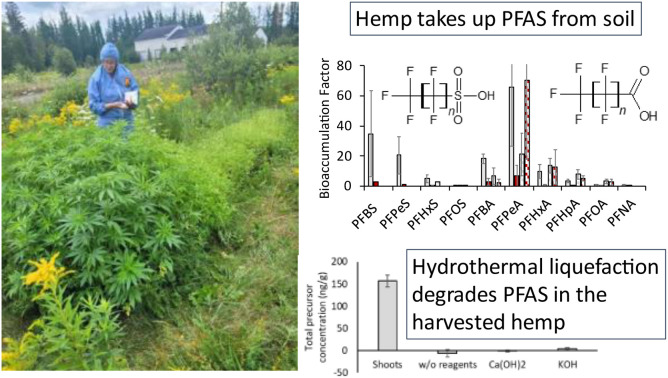

**Environmental significance**

Per- and polyfluoroalkyl substances (PFAS) are a class of recalcitrant, highly toxic contaminants, with limited remediation options. In this community-based field trial, we tested phytoremediation with hemp as a method to remove PFAS from soil, and hydrothermal liquefaction as a method for degrading PFAS in the harvested hemp. We identified 28 PFAS in soil and found hemp uptake of 10 of these PFAS, though the percentage of total PFAS removed from soil was low. Hydrothermal liquefaction successfully degraded several of the PFAS taken up by the hemp. While not a comprehensive PFAS solution, this project has had positive community impacts and lowered the overall presence of PFAS at this contaminated site.

## Introduction

Per- and polyfluoroalkyl substances (PFAS) are a class of highly toxic chemicals that encompasses thousands of compounds that contain extremely strong carbon–fluorine bonds. Very low exposure concentrations, in the parts per trillion range, can cause a variety of health effects including changes in cholesterol and thyroid hormone levels, as well as decreased response to vaccines.^1^ PFAS have been in use since the 1940s as ingredients in stainproof, greaseproof, and waterproof coatings, surfactants, and aqueous film-forming foams (AFFFs) used for firefighting.^2^ High levels of PFAS usage in many products has led to their widespread distribution in the environment.^3,4^ Due to their recalcitrant nature and the wide range of physicochemical properties of PFAS, remediation has proved to be extremely challenging.^5,6^ While an increasing number of options are available for removing PFAS from water,^7,8^ fewer are available for remediating soil.^5,6^ Phytoremediation of PFAS has begun to receive attention due to its low cost, potential for community engagement, and moderate levels of success with other contaminant classes.^9–12^

There are multiple approaches to phytoremediation. Plants can be used to degrade, stabilize, extract, or volatilize contaminants from soil.^13^ Here, the goal is phytoextraction, where PFAS are taken up into plant shoots that can subsequently be removed from the site. PFAS are accumulated by a wide range of plant species, though there is some variability.^10,14^ Fiber hemp was chosen for this study as it is an annual crop that grows quickly, takes up large amounts of water, has limiting grazing by animal species, and does not shed substantial leaf matter back into the soil. As plants and the bacteria associated with them are typically not able to degrade C–F bonds,^5^ PFAS removed from the soil by hemp are likely to retain the toxic fluorinated portion of their structure. A potential advantage of using fiber hemp for this work is that the parts of the plant that are less susceptible to bioaccumulation of PFAS (stems) may be able to be used in products such as bricks and rope. However, there is currently minimal information available about the specific location of PFAS within exposed hemp plants. Alternatively, contaminated hemp may be used for fuel production through hydrothermal liquefaction (HTL), which has previously been shown to degrade PFAS in sewage sludge and *Typha latifolia*, a wetland plant.^15–20^

Connecticut Agricultural Experiment Station (CAES) scientists have previously worked with community members from the Mi'kmaq Nation (Aroostook County) and Upland Grassroots (a community organization) to characterize soil and analyze hemp plants (grown by community members) at a site contaminated with AFFF at the former Loring Airforce Base in northern Maine, USA, which is now Mi'kmaq Nation land.^9,21^ Here, results are presented from a community initiated, field-scale phytoremediation trial at Loring Airforce Base, where both traditional targeted analysis and non-targeted analysis^21–23^ were used to quantify PFAS in soil and plants, as well as to examine the behavior of additional PFAS, including precursor compounds. Field-grown hemp was used in an HTL process designed to eliminate PFAS and produce fuel, and the products were tested to assess PFAS removal. Targeted and non-targeted analysis strategies were employed on the HTL products, as well as the total oxidizable precursor assay and extractable organic fluorine measurements to examine degradation of additional PFAS. To our knowledge, this is the first phytoremediation study to employ both targeted and non-targeted methods to examine PFAS. A flow chart of project activities and locations is shown in Fig. S1.†

## Methods

### Hemp growth and field sampling

Field trials were conducted at the former Loring Air Force Base in northern Maine, USA at the burn house site that was previously used for firefighter training. Our previous work identified over 90 potential PFAS in soil at this location, including concentrations of PFOS up to 152 ng g^−1^.^21^ Hemp was grown in 5 plots (Fig. S2†), including one near the drainage area where PFAS were measured in our previous work^21^ and four on higher ground on a man-made berm that surrounds the parking lot. Four varieties of hemp were tested: ChinMa (purchased from Hemp Warehouse), H-51, Hliana, and Hlesila (purchased from Rohrer Seeds). Each variety was grown in a subsection of each plot. Each plot was 1.2 m × 6 m and sub-plots were 1.2 m × 1.5 m. ChinMa seeds were sown May 30, 2022, the other three varieties were sown June 16, 2022, and all hemp was harvested August 22, 2022. Quoddy Blend Lobster Compost (advertised as PFAS free) was applied to hemp plots during planting, and the hemp was fertilized with organic fish oil diluted in water in July 2022. Hemp was irrigated with well water from Littleton, Maine approximately every 10 days throughout the growing season. The compost, fish oil, and well water were not tested for PFAS. Soil samples were taken from the top 15 cm during planting and harvesting using stainless steel equipment rinsed with the irrigation water between samples. Control soil was taken from an area at the Burn House site where hemp was not planted. Field blank soil was collected off site using the same equipment used at the study site. Two hemp and two soil samples were taken for each hemp variety in each plot. Hemp samples were air dried prior to distribution to labs and stored at room temperature. Soil was stored in HDPE bottles at room temperature. Community members from the Mi'kmaq Nation and Upland Grassroots were the primary personnel who conducted this portion of the project, including growth plot site selection, planting, irrigating, growing, and harvesting of hemp plants, and soil sample collection.

### Hydrothermal liquefaction of hemp

Hemp variety ChinMa was used to test HTL as a method to degrade PFAS taken up by the hemp plants. Hemp stems and leaves from several growth plots were composited, homogenized, and divided into samples for analysis and for HTL. Hydrothermal liquefaction of hemp tissues was performed in 15 mL reactors (High Pressure Equipment Co. Erie, PA, USA) and run in triplicate. Dried hemp shoots (0.5 g) and 9.5 mL of deionized water with or without a reagent (*i.e.*, 5 mmol of Ca(OH)_2_, 10 mmol of KOH) was loaded into the reactor. The reactor was then sealed and heated at 300 °C for 2 hours. The concentration of the base reagent and the HTL conditions were based on our previous publications on destructing PFAS in *Typha latifolia*.^19,20^ After cooling down to room temperature, the HTL products were flushed out using 20 mL MTBE. The MTBE fraction was then evaporated under a fume hood.

### Sample preparation and targeted PFAS analysis

Hemp (leaves and stems) and soil samples corresponding to each subdivided field plot were prepared and analyzed at CAES. HTL products and a composite sample of hemp shoots used for HTL were analyzed at SUNY Albany. A subset of samples prepared in Albany were also analyzed at CAES to ensure comparability of results (Fig. S10†). Details of all sample preparation and instrumental methods are available in ESI Sections S1.1.2 and S1.1.3.† Similar to previous work,^21^ soil and hemp samples at CAES were homogenized, extracted three times with 400 mM ammonium acetate in methanol, evaporated under N_2_, and cleaned up using graphene carbon black. Isotope dilution was used for quantification. Analysis for hemp variety ChinMa and corresponding soil was completed on an Ultimate 3000 ultra-performance liquid chromatograph (UPLC) coupled with a Q-Exactive Orbitrap mass spectrometer (Thermo Scientific) with negative electrospray ionization in FullMS-ddMS2 mode with additional all ion fragmentation scans. Use of the Orbitrap mass spectrometer allowed for non-targeted analysis of these samples. Remaining samples were analyzed using an Agilent 1290 UPLC coupled with a SciEx 7500 triple-quadrupole mass spectrometer, for targeted analysis only. A subset of samples were run on both instruments to demonstrate consistency of results (ESI Section 1.1.5†). Bioaccumulation factors were calculated by dividing concentrations in the plant (ng g^−1^) by concentrations in the soil (ng g^−1^). Reporting limits were 0.02 ng g^−1^ in soil and 0.05 ng mL^−1^ in hemp extracts, which corresponded to approximately 0.4 ng g^−1^ dry weight in hemp. Data below the reporting limits are not included in any averages or statistical analyses. We used hemp PFAS concentrations (targeted analytes only) to estimate the total amount of PFAS removed from the site in the 2022 growing season. Details can be found in ESI Section S1.1.7.†

Hemp samples analyzed in Albany were extracted according to a previously developed procedure.^24–26^ Briefly, the freeze-dried plant samples were pretreated with NaOH (0.4 M), tetrabutylammonium hydrogen sulfate (TBAHS, 0.5 M), and Na_2_CO_3_ buffer (0.25 M), sequentially, then extracted three times with *tert*-butyl methyl ether (MTBE). The MTBE extracts from 3 rounds of extraction were combined, evaporated under N_2_, reconstituted in 1 mL of methanol, and diluted with 9 mL of water in sequence. The sample was then subject to solid phase extraction (SPE) using a HyperSep C18 cartridge (Thermo Scientific). All analyses were run in triplicate. HTL products were air-dried and subject to PFAS extraction following EPA draft method 1633.^27^ The extracts of hemp shoots and HTL products were separated into 3 portions evenly. One portion was used for PFAS targeted analysis. Another portion was further processed with a total oxidizable precursor (TOP) assay. The last portion was used for extractable organic fluorine analysis. Targeted analysis was carried out using an Agilent 6470 Triple Quad Mass Spectrometer (LC-MS/MS, Santa Clara, CA, USA). Details can be found in ESI Sections 1.2.1 and 1.2.3.†

### Non-targeted analysis

Non-targeted analysis (NTA) was performed using the data files collected on the Orbitrap mass spectrometer at CAES. PFAS annotation for non-targeted analysis (NTA) was completed using FluoroMatch Flow, version 3.2.^21–23,28^ ChinMa hemp stem and leaf samples grown in the drainage area growth plot and their corresponding fall and spring soil samples were included in the FluoroMatch analysis. Both extraction and instrument blanks were included, and blank filtering was performed. Annotated compounds were manually curated to ensure accuracy of identifications. Reported results include homologous series of 3 or more PFAS with increasing retention times where at least one annotation was supported by MS2 data, as well as any compounds identified as known PFAS using fragmentation data. All reported annotations are supported by isotope pattern matching in the MS1 spectra. Our annotations meet the requirements for level 3 on the Schymanski scale:^29^ we are confident in the molecular formula and compound class, though we do not have enough evidence to be sure of the exact structure (*e.g.*, branching pattern).

Semi-quantification of annotated compounds was performed using TraceFinder version 4.1. Annotated compounds were semi-quantified in all ChinMa hemp and corresponding soil samples, control soil, and hemp and HTL extracts provided by the Albany team. Peak integrations were manually curated to ensure accuracy. Calibration surrogates were used and chosen based on similarity of PFAS class and nearness of retention time (Table 1).^30,31^ Additional details are provided in ESI Section S1.1.6.†

**Table 1:** Non-targeted PFAS annotations

Abbreviation	Molecular formula	Mass	RT (min)	Calibration surrogate	Estimated soil concentration^a^ (ng g^−1^)	
					Spring	Fall
**Fluorotelomer carboxylic acids (FTCs)**						
5 : 3 FTC	C_8_H_5_F_11_O_2_	341.0045	12.09	PFDA	1.4	1.2
6 : 3 FTC	C_9_H_5_F_13_O_2_	391.0018	13.46	PFUdA	**0.8**	**0.5**
7 : 3 FTC	C_10_H_5_F_15_O_2_	440.9994	14.31	PFDoA	**10.8**	**6.9**

**Sulfonamides**						
PFBSA	C_4_H_2_F_9_NO_2_S	297.9593	9.39	PFOSA	**0.6**	**0.4**
PFHxSA	C_6_H_2_F_13_NO_2_S	397.9533	13.06	PFOSA	**16.1**	**12.2**

**Sulfones**						
6 : 4 FT-sulfone	C_11_H_9_F_13_O_4_S	482.9925	12.91	PFOS	2.1	3.4

**Pentafluorosulfides**						
PFOS-PeFS	C_8_HF_21_O_3_S_2_	606.8976	14.17	PFDS	10.5	9.8

a Average of soil concentrations from ChinMa growth plot in high PFAS area (*n* = 2). Bold numbers indicate a decrease > 20%.

### Total oxidizable precursor assay

The total oxidizable precursor (TOP) assay was used to quantify additional PFAS in hemp and HTL products to determine the effects of HTL on PFAS that were not included in the targeted analysis. Prior to the TOP assay, extracts were evaporated to dryness under nitrogen gas. The dried material was resuspended in 6 mL of deionized water containing 60 mM persulfate and 150 mM NaOH. The samples were then heated at 85 °C for 6 hours. After reaction, all samples were neutralized with HCl and subjected to solid phase extraction (SPE) using HyperSep C18 cartridges. After the TOP assay, precursors to both PFCAs and PFSAs are proposed to be converted to PFCAs.^32,33^ The concentration of precursors was calculated by subtracting the total concentration of PFCAs in the sample before TOP assay from the total concentration of PFCAs after TOP assay. Additional details are available in ESI Section S1.2.2.†

### Extractable organic fluorine analysis

Extractable organic fluorine was measured in HTL products and corresponding hemp shoot samples. The analysis of extractable organic fluorine was conducted using a Metrohm 930 Combustion Ion Chromatograph (CIC). Briefly, the last portion of PFAS extracts was concentrated to ∼200 μL under N_2_. The concentrated extract was then loaded to a combustion boat and burned at 1050 °C for 10 min. The extractable organic fluorine was then transformed to inorganic fluoride and quantified by the Metrohm 930 CIC.

## Results

### Plant growth

Only one variety of hemp grew well over the course of the growth season – ChinMa, which grew to 1.2 meters before starting to flower in late August. Approximately 18 kg of ChinMa hemp was harvested. The other hemp varieties H51, hlesia and hliana (collectively referred to as ‘small hemp’), which were planted 2 weeks after the ChinMa hemp but harvested at the same time, reached a height of approximately 0.3 meters before the harvesting date. Approximately 7 kg of small hemp was harvested. Example photos are provided in Fig. S5.† The limited growth observed for the H51, hlesia and hliana is potentially due to the photoperiod response promoting early flowering; these varieties may be better suited to climates where earlier planting is possible and latitudes with less drastic photoperiod shifts throughout the growth season. These varieties are likely well-suited for phytoremediation in locations amenable to their growth, as evidenced by the similar bioaccumulation results collected for all 4 hemp varieties (Fig. S9†).

### Soil characterization

**Fig. 1:**
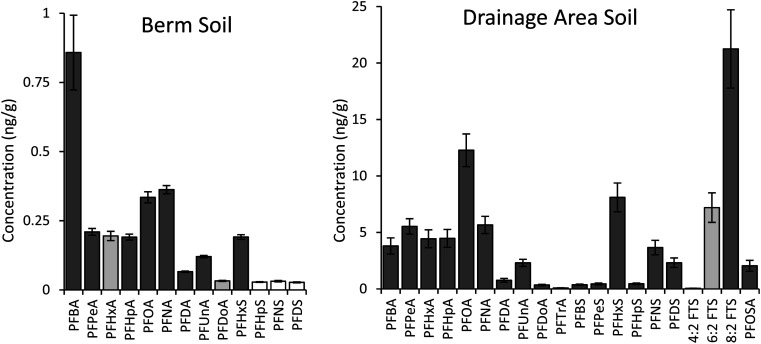
Fall concentrations of PFAS in field soils from berm and drainage area. Error bars represent standard error (berm soil 7 ≤ *n* ≤ 32, drainage area soil 6 ≤ *n* ≤ 8, non-detects not included in calculations). Bar color indicates detection frequency: dark gray 100%, medium gray 75–99%, and white below 74%. Data for PFOS is not shown (7.5 ng g^−1^ in berm soil and 107 ng g^−1^ in drainage area soil).

As in previous work,^21^ the growth plot closest to the drainage area had notably higher PFAS than the other four growth plots in the berm area. PFOS was the primary contaminant in all soil samples, at 107 ± 34 ng g^−1^ in the soil near the drainage area and 7.5 ± 1.3 ng g^−1^ in the berm growth plots. Twenty additional targeted PFAS were detected above the reporting limit of 0.02 ng g^−1^ in the drainage area soil, while 14 additional PFAS were detected in the berm soil (Fig. 1).

Soil concentrations were compared between fall and spring for growth plots where ChinMa hemp was grown and the control plot where no hemp was planted (Fig. S7 and S8†). There were no statistically significant decreases in concentrations for PFAS detected in both areas of hemp plots (paired *t*-tests, 1-tailed, all *p* ≥ 0.05). 6 : 2 FTS and 8 : 2 FTS were detected only in the drainage area, and soil concentrations decreased by greater than 35% in both replicates (Fig. S8†). Only two replicates were available for ChinMa hemp grown in high PFAS soil, so no statistical comparison was possible. 8 : 2 FTS was detected in control soil (*n* = 3), but no decrease occurred for 8 : 2 FTS or other detected PFAS (paired *t*-tests, 1-tailed, all *p* ≥ 0.05) (Fig. S7†). Due to lack of significant results for the ChinMa growth area, soil concentrations were not compared for small hemp plots. Fall soil concentrations are used in all subsequent analyses (including Fig. 1).

### PFAS accumulation by hemp

**Fig. 2:**
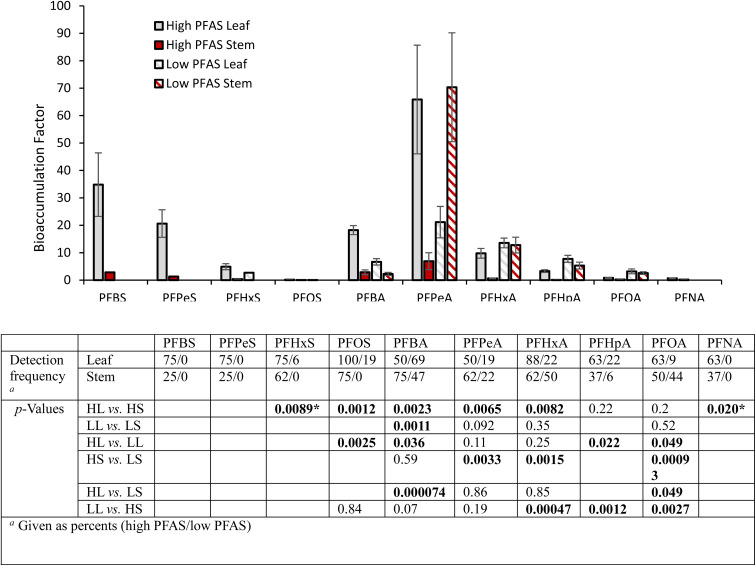
The bar graph shows bioaccumulation factors (all hemp varieties combined) for PFAS in hemp stems and leaves grown in low (berm) and high (drainage area) PFAS soils. All measurements above the reporting limit are shown. Error bars represent standard error for categories with at least 3 measurements (1 ≤ *n* ≤ 22, non-detects not included in calculations). The table shows detection frequencies and *p*-values comparing bioaccumulation factors for leaves and stems in low and high PFAS exposures. Statistically significant values are bolded (*α* = 0.05, Kruskal Wallis with Dunn's post-hoc analysis). A separate test was run for each PFAS. Values with a * are based on a *t*-test (2-tailed, unequal variance assumed), as only 2 values were compared. All categories with at least 3 measurements are included in the statistical analysis.

We detected 10 PFAS in hemp plants (Fig. 2). The data is reported as bioaccumulation factors, which are calculated by dividing the plant tissue concentration by the soil concentration for the same sub-plot. Bioaccumulation data is separated between hemp leaves and stems, as well as between the high (drainage area) and low (berm) PFAS growth plots. All compounds detected in at least 3 replicates in at least one sample category are included. No significant differences were found between bioaccumulation factors in small hemp and ChinMa hemp varieties or among small hemp varieties (Fig. S9†); consequently, data from all varieties is combined in Fig. 2. In general, our observations fall within the range of PFAS uptake reported for other plants.^14^ Bioaccumulation generally decreased with C–F chain length, though PFPeA had higher bioaccumulation than PFBA. The accumulation of carboxylic acids was typically higher than the sulfonic acids with the same number of carbons.

In the high PFAS growth plot, bioaccumulation in leaves was typically greater than stems. In the low concentration growth plot, only PFBA showed a significant difference between leaves and stems (leaves was higher), though statistical power was limited by low detection rates and high variability in measurements. For leaves, PFOS and PFBA had higher bioaccumulation in the high PFAS plot than in the low, while PFHpA and PFOA had higher bioaccumulation in the low PFAS plot. Stems had higher bioaccumulation in the low PFAS plot than in the high for PFPeA, PFHxA, and PFOA.

We estimate that the total PFAS mass taken up into above-ground hemp tissues and removed from soil was 1.4 mg (includes targeted analytes only). Approximately 85% of total removed PFAS mass was found in leaves, and approximately 75% of total removed PFAS mass was in the ChinMa hemp, though it only occupied 25% of the growth plot area. ChinMa hemp removed approximately 0.21 mg m^−2^ in the high PFAS soil near the drainage area, and approximately 0.09 mg m^−2^ in the lower PFAS berm soil, representing approximately 0.2% and 2.0% of the total soil PFAS respectively in the zone affected by hemp roots. Comparing individual compounds, PFPeA had the highest mass removed (0.79 mg), representing 56% of the total, and was the only compound where greater than an estimated 0.2 mg was removed. Exact calculations were not possible because only estimated masses were available for the total harvested hemp. Calculation details can be found in ESI Section S1.1.7.†

### Non-targeted analysis of hemp and soil

We identified 18 PFAS using our NTA workflow, including 11 compounds also investigated using targeted methods. Agreement between analytical strategies increases confidence in the annotations for compounds not included in targeted analysis, which are listed in Table 1. Additional annotation details are provided in Table S8.† Estimated concentrations are reported based on surrogate calibration curves. The absolute values derived from this method may be off by an order of magnitude or more, but the relative amounts reported within the data for a single compound are likely to show an accurate comparison.^30,31^ The same reporting limits were used as in the targeted analysis.

All 7 compounds were detected in both soil samples from the high PFAS plots where ChinMa hemp was grown in both spring and fall. There was a greater than 20% decrease in estimated concentration (*n* = 2) for 4 compounds, including 2 FTCs and 2 sulfonamides. In the low PFAS area, only PFHxSA and PFOS-PeFS were detected, with estimated concentrations averaging 0.03 ng g^−1^ and 0.07 ng g^−1^ respectively and detection frequencies of 56% and 75%, respectively. There were no decreases in average concentration greater than 20%. In control soil, where no hemp was grown, 7 : 3 FTC, PFHxSA and PFOS-PeFS were detected, with average estimated concentrations of 0.3 ng g^−1^, 0.2 ng g^−1^, and 0.7 ng g^−1^ respectively (detection frequencies 50%, 83%, and 100% respectively). 7 : 3 FTC was only detected in spring soil, while the others did not show statistically significant differences between spring and fall (*n* = 3, paired *t*-tests, one tailed, all *p* ≥ 0.05). PFBSA was detected in one ChinMa stem sample from the high PFAS area at an estimated 0.45 ng g^−1^. Other NTA compounds were not detected in hemp or in HTL products.

In our previous work on soil from Loring, we detected sulfonamides, sulfones, and pentafluorosulfides, as well as several additional classes of PFAS.^21^ It is not surprising that more classes of PFAS were detected in those samples, as they were taken from deeper in the drainage area of the site where the concentrations of targeted PFAS were also higher. We did not detect any fluorotelomer carboxylic acids in our previous work. It is possible that these compounds were not present in those samples, or that improvements in FluoroMatch libraries^23,28^ enabled their identification in the present study.

### Degradation of PFAS in hemp *via* hydrothermal liquefaction

**Fig. 3:**
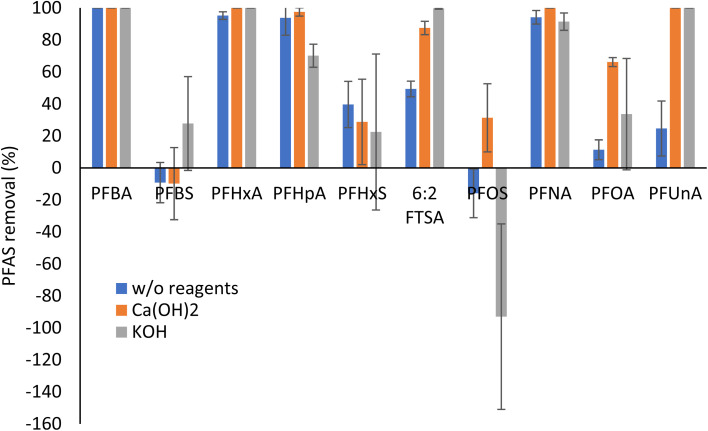
Mass removal and increase (%) of PFAS in hemp shoots after HTL with or without basic reagents (*n* = 3).

**Fig. 4:**
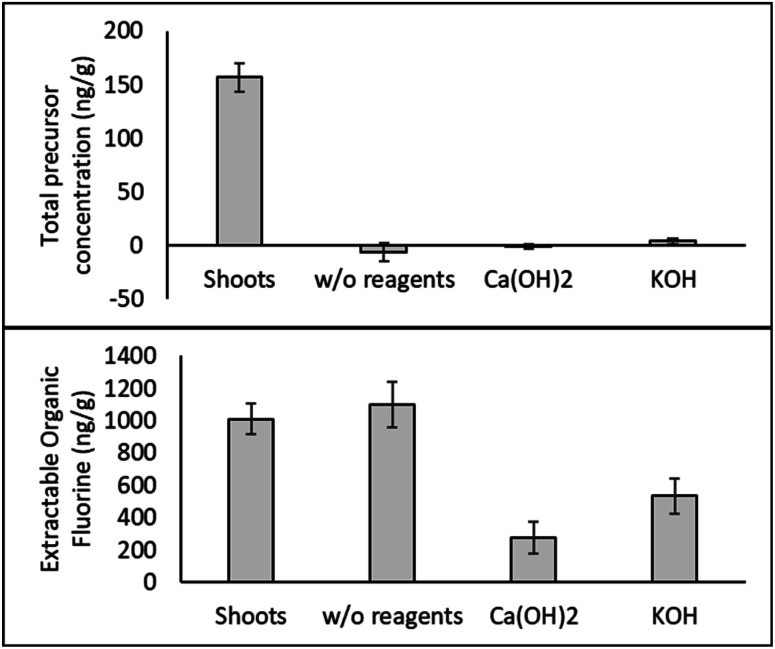
Concentration of total PFAS precursors (top graph) and extractable organic fluorine (bottom graph) in hemp shoots and products after HTL with or without basic reagents (*n* = 3).

As shown in Fig. 3, perfluorocarboxylic acids (PFCAs), including PFBA, PFHxA, PFHpA, and PFNA, were largely degraded after HTL, regardless of the presence of basic reagents. This was consistent with our previous observation that HTL at 300 °C for 2 hours effectively degraded PFCAs (>99%).^19^ In this study, the degradation of PFOA and PFUnA after HTL without any basic reagents was lower than other PFCAs. The addition of Ca(OH)_2_ or KOH remarkably improved the degradation of PFUnA, while only Ca(OH)_2_ significantly enhanced the removal of PFOA. Basic reagents, especially KOH, also largely improved the degradation performance of HTL for 6 : 2 FTS. Regarding perfluorosulfonic acids (PFSAs), the degradation was limited. Interestingly, there was a significant increase of PFOS mass in the HTL products after the thermal treatment, especially with KOH. Such mass increase could be due to the transformation of PFOS precursors to PFOS during HTL, though PFOS precursors were not detected in hemp using our NTA workflow. However, the TOP assay results showing changes of total PFAS precursors in hemp shoots after HTL (Fig. 4a) support this hypothesis.

Measurements of the extractable organic fluorine (EOF) give an idea of the amount of unidentified organic fluorinated compounds present in the samples. HTL with basic reagents substantially lowered EOF in hemp shoots, indicating that Ca(OH)_2_ and KOH significantly enhanced the defluorination efficiency of PFAS by HTL.

## Discussion

As found in our previous work,^21^ the soil at Loring Airforce Base contains a wide range of PFAS compounds that likely come from historical AFFF use. Based on the lack of significant differences between PFAS concentrations in spring and fall soil, phytoremediation with hemp is not a fast solution to PFAS contamination in soil. However, given the high bioaccumulation we saw for shorter chain PFAS, if grown over a period of years, decreases in soil concentrations are expected. We calculated that ChinMa hemp could remove up to approximately 2% of total PFAS in the area affected by hemp roots. The soil samples in this study only included the top 6 inches of soil, while hemp roots typically penetrate deeper into the ground. It is possible that the PFAS taken up by the hemp are coming from below our soil sampling range. For longer chain PFAS like PFOS, bioaccumulation was very low, and additional strategies will be necessary for remediation. However, our analyses did not include the hemp roots, as they would not typically be harvested as part of a hemp crop. Longer chain PFAS are known to accumulate more in plant roots,^14,34^ so harvesting roots may be more effective than stems and leaves for removing PFAS from the site. It is also possible that the phytoremediation helps to stabilize PFAS in the soil through sorption to plant roots and the associated organic matter from root exudates and rhizosphere bacterial community. Contaminants that are stabilized through sorption are less likely to contaminate groundwater or be taken up by plants.^13^ Further investigation is necessary to determine how hemp roots affect PFAS in the surrounding soil and the depth of soil affected by hemp roots.

In the targeted analysis, we found that bioaccumulation was the highest for smaller PFAS that are more hydrophilic. Our NTA results primarily feature larger compounds, with fairly late retention times that indicate high hydrophobicity. Correspondingly, only the lightest compound found using NTA was detected in hemp, though others also decreased in the soil.

While not detected in plants, our data shows evidence of enhanced degradation of PFAS precursor compounds in hemp plot soil. Both 6 : 2 FTS and 8 : 2 FTS decreased by greater than 35% in the ChinMa high PFAS growth plot, and four of seven non-targeted compounds decreased by greater than 20%. These changes were not seen in the control plot. All of these compounds contain headgroups that are amenable to biological degradation. Bacteria can play a crucial role in the degradation of persistent contaminants. Bacteria often found in the root zone of plants, have the ability to break down and detoxify these pollutants, contributing to the remediation of contaminated environments.^35,36^ In our study, it is likely that degradation occurred in the rhizosphere, helped by microbes associated with the hemp roots. It is also possible that the precursor compounds were taken up by the hemp and degraded *in planta*. Rhizosphere biotransformation of precursor PFAS is an important topic for future investigation.

The TOP assay and TOF results provide evidence that additional PFAS precursors were present in hemp samples but not identified *via* our NTA approach. Lack of detection of these compounds using NTA could be due to the differing hemp extraction methods used and the bias introduced by use of SPE, insufficient MS2 spectra collection during LC-HRMS analysis, and/or limitations in FluoroMatch, which relies heavily on detection of common PFAS fragments and homologous series.^23^ Future work comparing hemp extraction methods, using iterative approaches for MS2 spectra collection,^22,37^ and including other NTA identification strategies^21^ may provide more information on PFAS precursors in plant tissue. We also note that our detection methods lacked the capability to detect ultrashort chain PFAS, which may be present in both original and TOP assay samples.

For commercial products made from hemp, such as bricks and rope, the fibers in the stem are used, while the leaves are discarded. Therefore, higher bioaccumulation of PFAS in leaves for plants grown in the high PFAS area is a promising result for the potential industrial use of hemp stems grown on contaminated land. Hemp has two useful types of fiber in the stem: bast and hurd. Future research should characterize PFAS accumulation in these components separately, as well as on the fate of PFAS during industrial processing of hemp fibers.

The HTL results show potential for destruction of some PFAS taken up by hemp, though degradation of sulfonic acids is not complete, and not all of the extractable organic fluorine is degraded. Different from the finding in this study, our previous results showed that HTL without any basic reagents removed >99% of PFOA (>99%) and 49.7% of PFOS in *Typha latifolia* (cattail plants).^19^ However, the cattail plants for HTL in the previous study were only exposed to five PFAAs in a hydroponic system. There were no other PFAS taken up by the plants and could potentially transform to targeted PFAAs. We hypothesize that the presence of PFAS precursors in this study led to decreased HTL degradation efficiency and increased need for base catalyzation of the process. Wu *et al.*^38^ also reported that NaOH and other reagents that increase pH can promote defluorination of PFAS, such as PFOS. The authors proposed that OH- could catalyze the cleavage of the sulfonate headgroup of PFOS, followed by rapid sequential decarboxylation reactions, eventually leading to complete mineralization of PFAS.^38^ Additional investigation of HTL degradation of complex PFAS mixtures is warranted.

## Community significance and conclusions

While there are currently limitations for phytoremediation of PFAS as the primary strategy for mitigating PFAS contamination, the current findings provide valuable understanding about this method. It is currently estimated that the safe planetary boundary for PFAS has already been exceeded, and without advances in remediation technology, PFAS will continue to cycle through the environment at toxic levels indefinitely.^39^ Finding solutions for this is imperative for members of the Mi'kmaq Nation and Upland Grassroots, who care deeply about the land as well as their personal potential exposure to contaminants, and want to find safe and sustainable solutions to speed up the timeline for cleaning PFAS from the environment for the sake of future generations and the natural world. Pursuing phytoremediation solutions in the face of the currently limited options is an obvious approach that can make a difference in PFAS that are already present. Phytoremediation can also be a good way to get community members engaged in solving environmental problems. Even small improvements can be a significant achievement and can draw attention to problems that require funding and attention from government and industry. This project already has attracted participation from many community members, drawn the attention of both local and online press,^40–42^ resulted in discussion with potential collaborators worldwide with interest in implementing phytoremediation in their own communities, and led to the submission of many small grant proposals and one larger one recently submitted to the US EPA, all of which have prominently featured community outreach and activities. While improvements in the local levels of PFAS have been modest, every molecule of PFAS taken up by a plant and removed from the site results in less PFAS free in the environment.

Future investigations should continue to examine effects of phytoremediation and HTL on PFAS precursors and seek out methods for improving plant uptake of longer chain, larger PFAS molecules. Additional investigation is also warranted for sites with high levels of short-chain PFAS contamination, where phytoremediation may be an important strategy to remove and reduce mobility of these hydrophilic compounds. While not yet optimized, phytoremediation is a community-friendly method of making a difference in PFAS contamination and should receive continued study.

## Author contributions

SLN: conceptualization, data curation, formal analysis, funding acquisition, investigation, methodology, project administration, resources, supervision, validation, visualization, writing – original draft, writing – review and editing; ST: investigation, methodology, supervision, validation, writing – review and editing; CS: conceptualization, investigation, methodology, project administration, resources, writing – review and editing; RS: conceptualization, investigation, project administration, resources; MB: investigation; WZ: formal analysis, investigation, methodology, validation, visualization, writing – review and editing; YL: project administration, resources, supervision, writing – review and editing; JJ: investigation; NZ: conceptualization, funding acquisition, project administration, supervision, writing – review and editing; JCW: funding acquisition, resources, supervision, writing – review and editing; CLH: funding acquisition, writing – review and editing; VV: funding acquisition, writing – review and editing; BB: conceptualization, funding acquisition, investigation, project administration, resources, writing – review and editing; MT: conceptualization, funding acquisition, investigation, project administration, resources, writing – review and editing.

## Conflicts of interest

BB is affiliated with Rohrer seeds, where some varieties of hemp seeds were purchased.

## Supplementary Material

VA-003-D3VA00340J-s001

VA-003-D3VA00340J-s002
