# Supplementary material for: A comprehensive trial on PFAS remediation: hemp phytoextraction and PFAS degradation in harvested plants
Source: Env Sci Adv. 2024 Jan 5;3(2):304–13. doi: 10.1039/d3va00340j (PMC10841816; doi:10.1039/d3va00340j)
Supplement: VA-003-D3VA00340J-s002 [file VA-003-D3VA00340J-s002.pdf]

Electronic Supplementary Material (ESI) for Environmental Science: Advances.
This journal is © The Royal Society of Chemistry 2024

Supporting Information:

A Comprehensive Trial on PFAS Remediation: Hemp Phytoextraction and PFAS Degradation in Harvested Plants

Sara L. Nason,[1]* Sara Thomas, [1] Chelli Stanley,[2] Richard Silliboy, [2,3] Maggie Blumenthal, [2] Weilan Zhang,[4] Yanna Liang, [4] Jasmine P. Jones, [1] Nubia Zuverza-Mena, [1] Jason C. White, [1] Christy L. Haynes,[5] Vasilis Vasiliou,[6] Michael P Timko[7], Bryan W Berger[8]

1. The Connecticut Agricultural Experiment Station, New Haven, CT 06511, USA
2. Upland Grassroots, Limestone, ME 04750, USA
3. Mi'kmaq Nation, Presque Isle, ME 04679, USA
4. Department of Environmental and Sustainable Engineering, University at Albany, State University of New York, Albany, NY 12222, USA
5. University of Minnesota, Minneapolis, MN 55455, USA
6. Yale School of Public Health, Department of Environmental Health Sciences, New Haven, CT 06510.
7. University of Virginia, Department of Biology, Charlottesville, VA 22903
8. University of Virginia, Department of Chemical Engineering, Charlottesville, VA 22903

*Corresponding author: Sara Nason: sara.nason@ct.gov

S1. Supplemental Methods

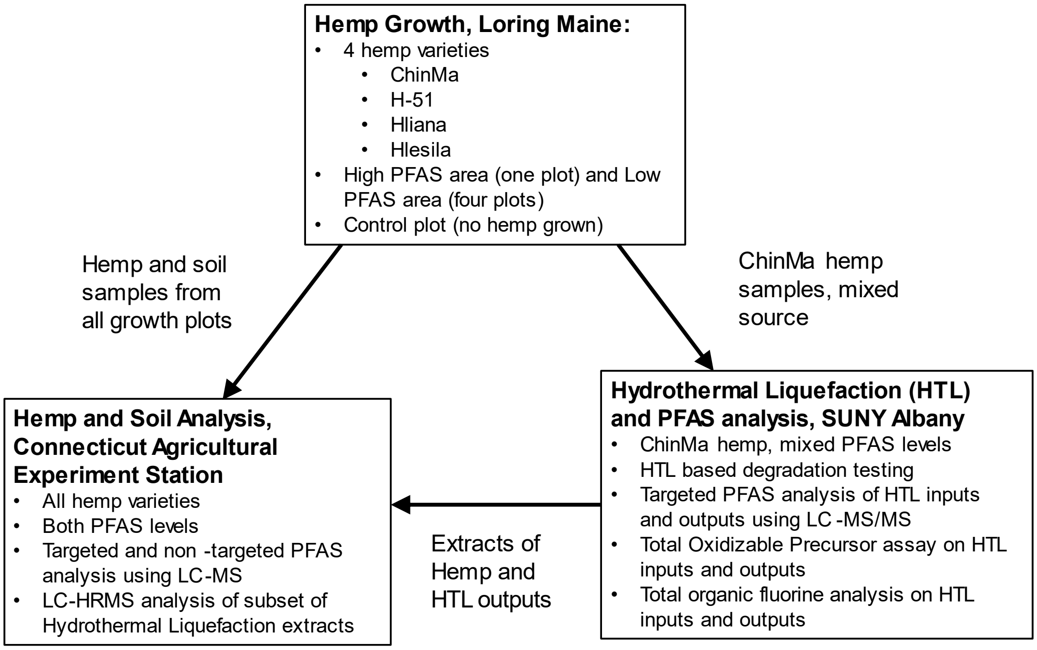

Figure S1. Flowchart showing project locations and activities. The hemp growth team in Loring Maine consisted primarily of community members from the Mi'kmaq tribe and Upland Grassroots, who were advised by scientists from multiple institutions.

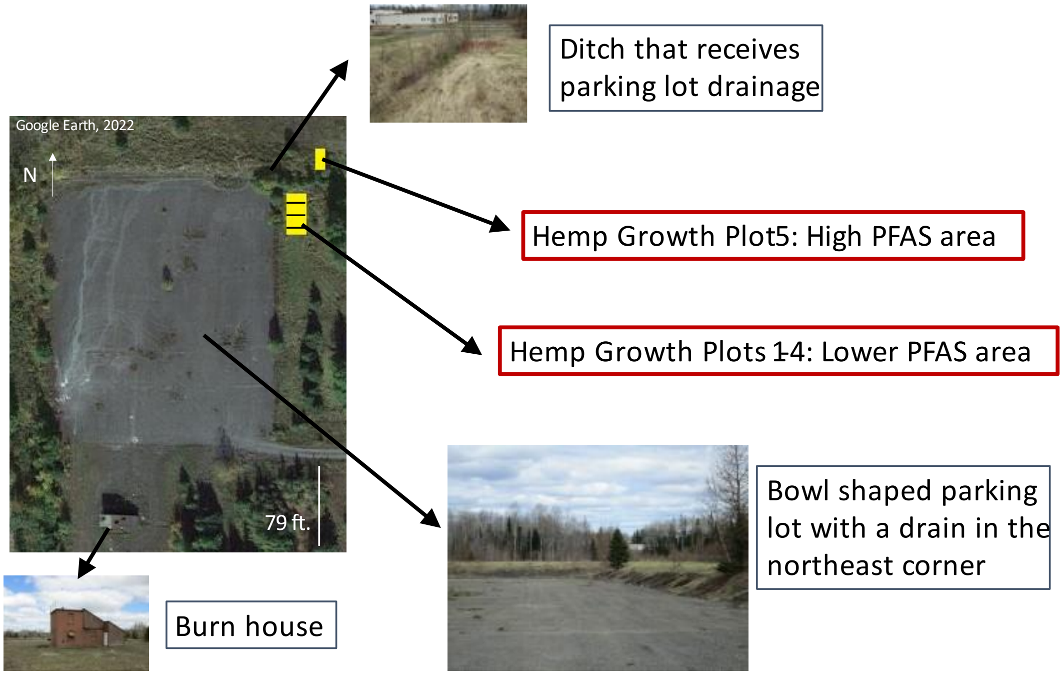

Figure S2. Diagram showing hemp growth plot locations relative to site features. Photo credit: Chelli Stanley

S1.1. Connecticut Agricultural Experiment Station Analytical Methods

S1.1.1. Materials

A 24 PFAS standard mixture (PFC-24) was purchased from Accustandard (New Haven, CT). A mixture of C-13 labeled PFAS was purchased from Wellington Labs. Included PFAS and their abbreviations are shown in **Table S1**. Solvents used were HPLC or LC-MS Optima grade and obtained from Fisher Scientific. Optima grade formic acid was obtained from Fisher Scientific. Ammonium acetate (ACS reagent grade) and Supelclean ENVI-Carb 120/400 was obtained from Sigma Aldrich. Ultrapure water was obtained from an in-house Milli-Q Integral 5 water purification system. Sample preparation used only polypropylene containers and pipette tips. Sample filters (0.2 µm) were made of regenerated cellulose and polypropylene and were obtained from Fisher Scientific (centrifuge filters) and Agilent (syringe filters).

S1.1.2. Sample Preparation

The extraction protocol was based on our previous work and Munoz et al., 2018 and was designed primarily for non-targeted analysis of a wide breadth of PFAS rather than for accurate quantification of a few.[1,2] Adaptations were made to the method in our previous work to include additional 13C labeled standards (listed in **Table S1**) and to accommodate hemp leaf and stem samples.

Soil samples were homogenized in a ceramic mortar and pestle then passed through a No. 16 1.18 mm bronze sieve. Hemp leaf samples were homogenized using a ceramic motor and pestle whereas the hemp stems were finely chopped by knife/scissors for sampling.

For soil, 2.00 g were extracted for each sample. For hemp, 0.5 g were extracted when available, but lower masses were used when not enough material was available. All samples were

spiked with the 13C PFAS mixture at a level of 0.5 ng/mL in the final extract for soil and 1 ng/mL in the hemp samples, and were equilibrated overnight prior to extraction. Samples were extracted three times with 4.00 mL of methanol containing 400 mM ammonium acetate. Each extraction consisted of 5 minutes of vigorous shaking on a paint can shaker followed by 5 minutes centrifugation at 3000 rpm. Supernatant from the three extractions was combined and evaporated under N_2 in a 60 °C water bath, then reconstituted up to 1 mL with methanol and vortexed. Extracts were transferred to polypropylene tubes containing 40 ± 5 mg of ENVI-Carb and vortexed followed by centrifugation at 14,000 rpm for 30 minutes. Supernatant was filtered through a 0.2 µm regenerated cellulose membrane. Equal volumes of extract and ultra-pure water in were combined in polypropylene autosampler vials, then analyzed by LC-MS. One solvent blank and one solvent spike (no soil or plant matrix) containing PFC-24 standard (components listed in **Table S1**) were extracted alongside each batch of samples.

S1.1.3. Instrumental Analysis

Chromatography was performed using a Thermo Ultimate 3000 (Thermo Q-Exactive samples) or an Agilent 1690 (SciEx 7500 samples) ultra-high performance liquid chromatograph (UPLC) equipped with a PFAS delay column and a Thermo Hypersil Gold C-18 column (100 mm x 2.1 mm, 1.9 µm particles) with an Accucore aQ guard column (10 mm x 2.1 mm, 2.6 µm particles). Mobile phases were 0.1% formic acid in ultra-pure water (A) and 0.1% formic acid in acetonitrile (B). Injection volume was 10 µL (Ultimate 3000) or 2 µL (Agilent 1690) and flow rate was 300 µL/min. The column oven was kept at 40 °C and the autosampler at 10 °C. The solvent gradient is provided in **Table S2**. Retention times were similar between instruments and are provided in **Table S1**. Negative electrospray ionization was used. Calibration range was 0.01 to 300 ng/mL. All standards contained the same 13C PFAS concentrations as the samples for each run. Every 10 to

15 samples, a solvent blank and a standard solution were analyzed to track instrument performance.

The Thermo Q-Exactive method included full MS scans, data dependent MS/MS (ddMS2) scans, and all ion fragmentation (AIF) scans within one injection (scan settings and source parameters in **Tables S3-S6**). Quantitative analysis was performed in TraceFinder 4.1 (Thermo) using FullMS scans. Calibration curves were weighted 1/x. Automated Genesis peak integration was used (9 smoothing points) and integrations were manually curated to ensure accuracy.

The SciEx 7500 triple quadrupole method settings are provided in **Table S7**. The instrument method was a scheduled MRM, allowing for many ions to be detected within a single run while maximizing dwell time for each ion. Quantitative analysis was performed using SciEx OS. Calibration curves were weighted 1/x. Automated MQ4 peak integration was used and integrations were manually curated to ensure accuracy.

Further quantitative analysis was performed in Microsoft Excel 365. Outlier data was removed from the hemp bioaccumulation dataset. Outliers were defined as data points greater than 2 standard deviations away from the mean (calculated separately for each PFAS).

S1.1.4. Extraction Recovery

Method recoveries were determined for each matrix (**Figure S3**). Percent recovery was calculated according to Equation S1:

$$\text{Percent Recovery}(\%) = \frac{C_{m,s} - C_{m,u}}{C_e} \times 100 \qquad \text{(Equation S1)}$$

Where $C_{m,s}$ is the measured concentration in the spiked sample, $C_{m,u}$ is the measured concentration in the unspiked sample, and C_e is the expected concentration.

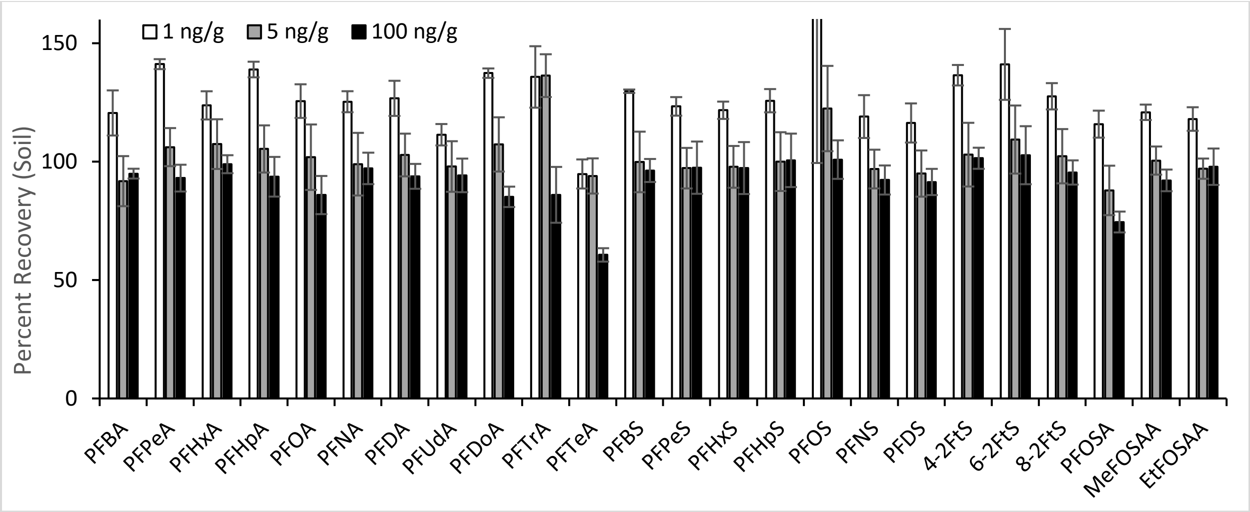

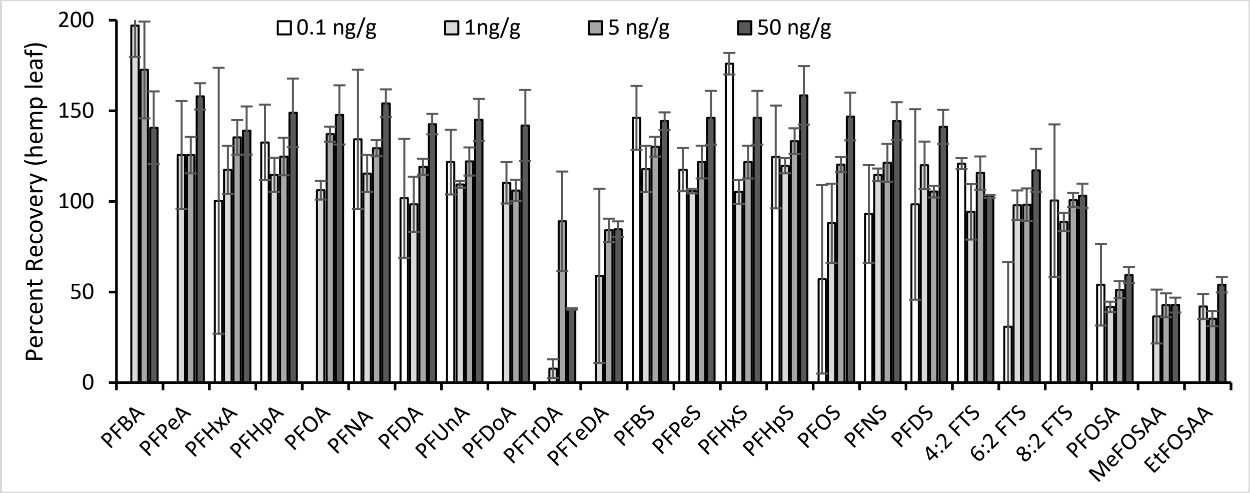

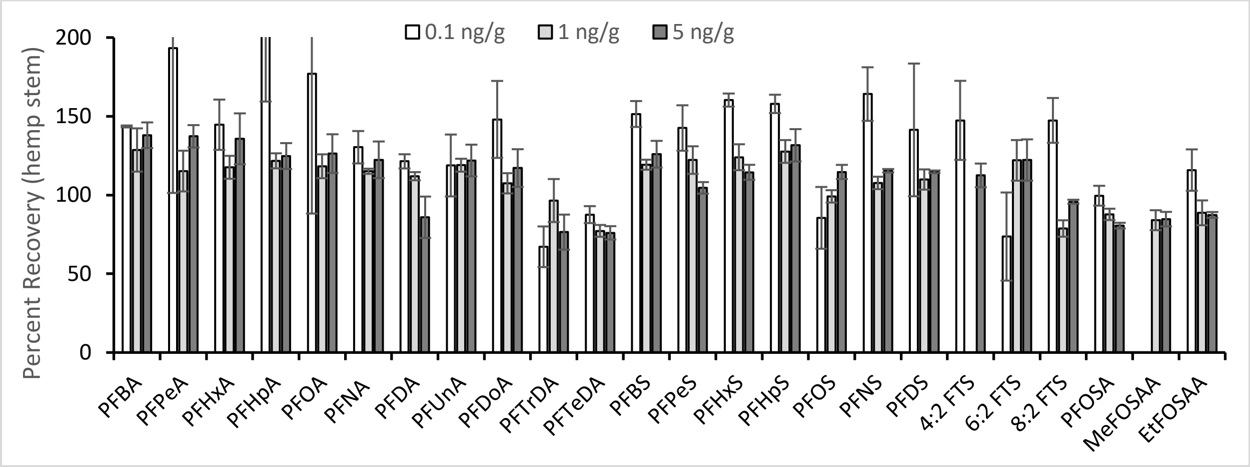

Figure S3. Extraction recovery in soil, hemp leaves, and hemp stems.

Extraction recovery was very consistent in soil. Testing at lower concentrations was performed, but results were poor due to high background levels of PFAS in the tested soil (most PFAS >0.2 ng/mL). Some signal enhancement was present in the hemp recovery samples, but consistency between replicates and across the concentration range was good. As in previous work, recovery was lower for hydrophobic PFAS.[1] If better accuracy is needed for future work, clean-up using weak anion exchange solid phase extraction (as in proposed EPA method 1633) should be pursued for hemp samples.

S1.1.5. CAES Instrument Comparison

Five soil, hemp leaf, and hemp stem (variety ChinMa) samples from growth plot 5 were analyzed using both LC-HRMS and LC-MS/MS at CAES. A comparison of results is shown in **Figure S4**. While there were some systematic differences between analyses, they are small relative to the variability amongst the samples. PFOS is excluded from the soil graph, but had measurements ± standard deviation) of 96 ± 32 ng/g using LC-MS/MS and 103 ± 36 ng/g using LC-HRMS. The same extracts and calibration samples were used in each analysis.

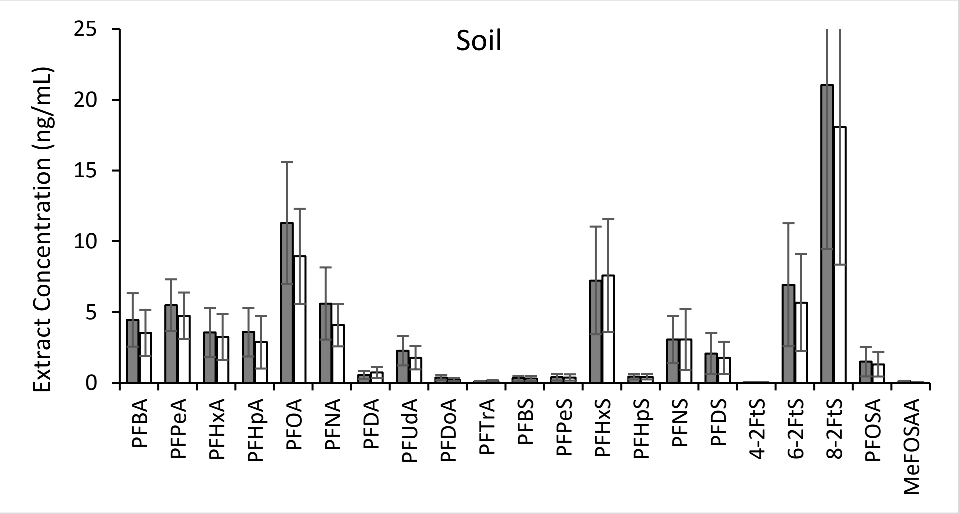

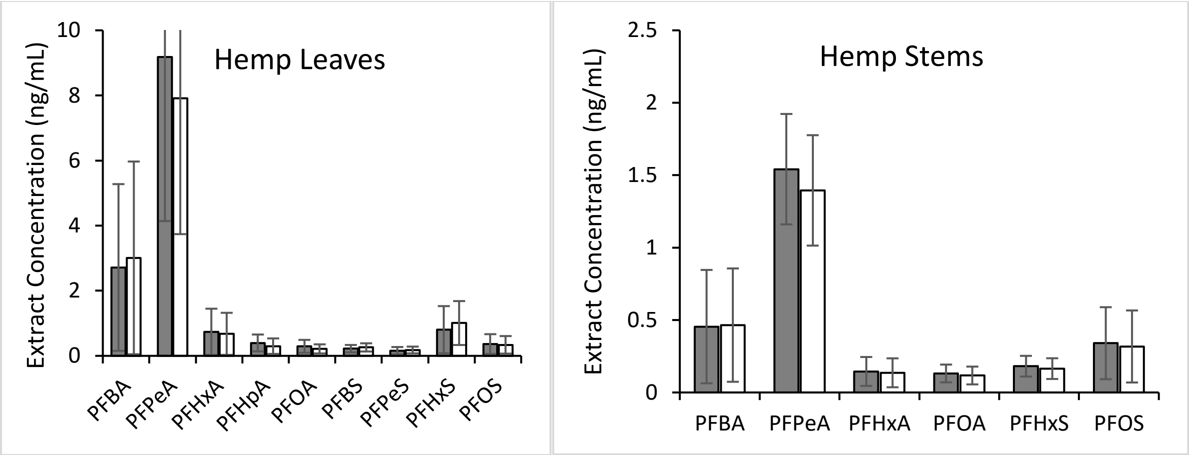

Figure S4. Average measurements for samples using LC-MS/MS (dark bars) and LC-HRMS (white bars). Error bars show standard deviation (n = 5).

S1.1.6. Non-Targeted Analysis

FluoroMatch Flow version 3.2 was used for non-targeted PFAS annotation.[3] Eight MS/MS data files were used: fall and spring soil from hemp plot 5, hemps stems from plot 5, and hemp leaves from plot 5 (2 replicates each). The same samples were used for MS1 analysis. A 100 ng/mL standard of the targeted analytes was also included in the analysis to help verify NTA results. Four target files were used, including fall and spring soil, leaves, and stems from subplot 5-1. Two extraction blanks and an instrument blank were used for blank filtering. Blank filtering used Equation S2:

$$A > 2*(B+(3*B_{\sigma})) \qquad \text{(Equation S2)}$$

Where A is the peak area required to be not be excluded by blank filtering, B is the average peak are in the blanks, and B_{σ} is the standard deviation of the peak area in the blanks. For peak picking, we used an MS/MS intensity threshold of 1000, a Full-Scan intensity threshold of 5000, and MS1 m/z search tolerance of 0.005 Daltons, and an MS/MS m/z search window of 10 ppm.

The only annotation results reported include homologous series of 3 or more PFAS with increasing retention times where at least one annotation was supported by MS2 data, and PFAS present in FluoroMatch libraries or the EPA master list identified in our samples using fragmentation data. Due to the complex sample matrices, and high noise level, the less confident identifications output by FluoroMatch were not manually investigated or included here.

PFAS identified via NTA were added to a compound database in TraceFinder 4.1 (Thermo Scientific). All ChinMa hemp and corresponding soil samples, control soil, and hemp and HTL extracts from the Albany team were semi-quantitatively analyzed for the NTA compounds, based on the masses and retention times found in FluoroMatch analysis. Each NTA compounds was assigned a calibration surrogate for semi-quantitation, as described in the main text (**Table 1**). The same calibration samples were used for NTA analytes as were used for targeted quantitation in each batch of samples.

S1.1.7. PFAS Mass Removal Calculations

We estimated the total PFAS mass taken up into above-ground hemp tissues and removed from soil. Small hemp and ChinMa hemp were considered separately. For each hemp compartment (e.g. ChinMa hemp stems), we multiplied the average concentration of each PFAS by the detection frequency and by the amount of hemp mass harvested. We assumed that harvested hemp mass was 50% leaves and 50% stem tissue. Totals for individual PFAS were summed to get a complete PFAS removal estimate for the 2022 hemp growth season. To calculate percentage of PFAS removal, we calculated total soil PFAS, assuming a soil depth of 0.5 m, affected area equivalent to the growth plot area, and average PFAS concentrations equivalent to those measured in surface level soil.

S1.2.: SUNY Albany Analytical Methods

S1.2.1. Plant extraction

The hemp shoots were vacuum dried at -37 °C for 48 hours, then ground to a homogenized powder/fiber mixture using a coffee grinder. The dried tissues were extracted according to a previously developed procedure.[4–6] Briefly, each dry hemp sample was spiked with 10 ng of $^{13}C_2$-PFHxA as the surrogate and mixed with 4 mL of NaOH (0.4 M) in a 50-mL polypropylene (PP) tube. After incubating at 4 °C overnight, 2 mL of tetrabutylammonium hydrogensulfate (TBAHS, 0.5 M) and 4 mL of Na_2CO_3 buffer (0.25 M) were added into the tube. Afterwards, 5 mL of tert-Butyl methyl ether (MTBE) were added to the mixture, followed by vigorous shaking for 20 min. The MTBE layer was then separated from the aqueous layer by centrifugation and transferred to a new PP tube. The plant residual was further extracted twice with MTBE. The MTBE extracts from 3 rounds of extraction were combined, evaporated under N_2, reconstituted in 1 mL of methanol, and diluted with 9 mL of water in sequence. The sample was then subject to solid phase extraction (SPE) using a HyperSep C18 cartridge (Thermo Scientific, Waltham, MA, USA), conditioned with 10 mL of methanol and 10 mL of MTBE. PFAS in the cartridge was eluted by 4 mL of methanol and 4 mL of 0.1% NH_4OH in methanol. All experiments were run in triplicate.

S1.2.2. Total oxidizable precursor assay

Prior to TOP assay, the extracts were evaporated to dryness under nitrogen gas. The dried material was resuspended in 6 mL of deionized water containing 60 mM persulfate and 150 mM NaOH. The samples were then heated at 85 °C for 6 hours. After reaction, all samples were neutralized with HCl and subjected to solid phase extraction (SPE) using HyperSep C18 cartridges, conditioned with 4 mL of 0.1% NH_4OH in methanol and 4 ml of water. PFAS were then eluted with 2 mL of methanol, followed by 2 mL of 0.1% NH_4OH in methanol.

S1.2.3. PFAS quantification

Quantification of PFAS in the extracts was carried out using an Agilent 6470 Triple Quad Mass Spectrometer (LC-MS/MS, Santa Clara, CA, USA). Before analysis, samples were spiked with $^{13}C_4$-PFOS and $^{13}C_2$-PFOA as internal standards following EPA Method 537.1 Rev 2. An Agilent ZORBAX Eclipse Plus C18 (3.0 × 50 mm, 1.8 μm) was used the analytical column at 50 °C. A binary mobile phase (solvent A: 5 mM ammonium acetate in water; solvent B: 5 mM ammonium acetate in 95% methanol) was applied and the flow rate was 0.5 mL/min. The mobile phase gradient profile started at 70% of A, decreased to 0% of A at 8 min and held for 4 min before reverting to original conditions. Other parameters and working conditions of LC-MS/MS were listed in our previous publication .[7] The extraction efficiency for PFAS in hemp shoots was determined by calculating the ratios of surrogate mass determined in samples to the initial spiked surrogate mass.

S2. Supplemental Results

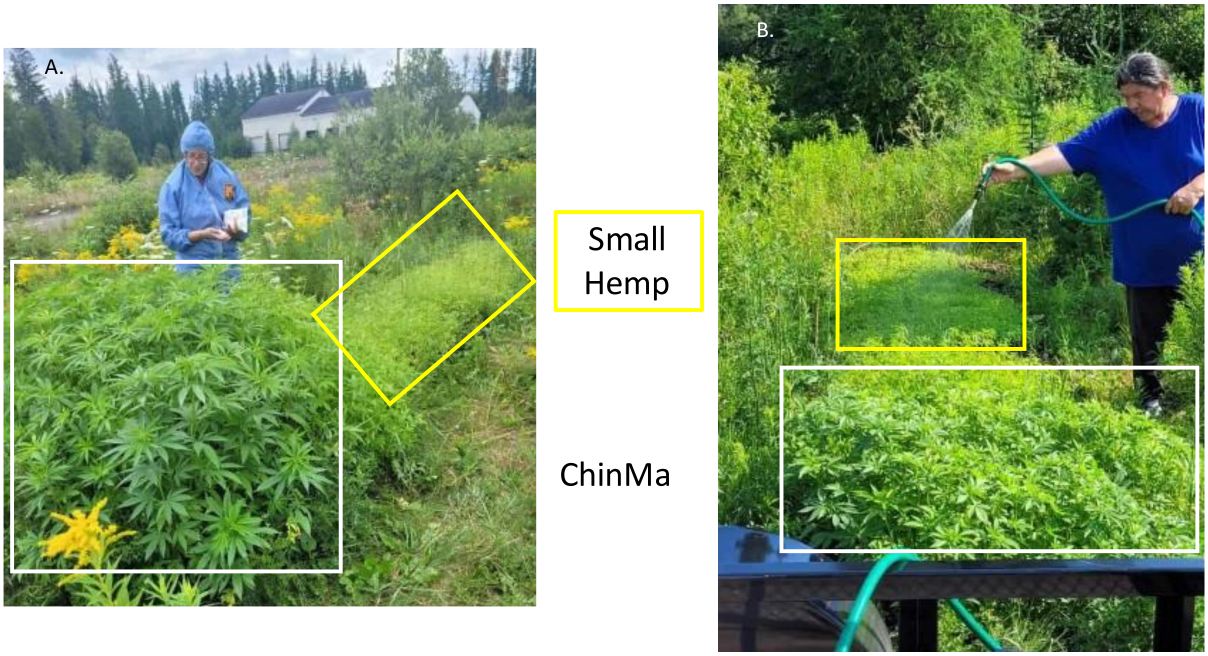

Figure S5. Photographs of hemp growth. ChinMa hemp (white boxes) is significantly larger than the small hemp varieties (yellow boxes) A. Chelli Stanley monitoring hemp growth in Plot 5. Photograph taken (August 11, 2022). Community member Maynard Marshall watering hemp plants in Plot 5. Photograph taken (July 16, 2022). Photography credit: (Richard Silliboy)

Field blank soil was collected at a location with no know PFAS contamination before (Blank-1) and after (Blank-2) spring soil sampling at the phytoremediation site, using the same equipment. Though the concentrations of PFAS in field blanks overlaps with the lower concentration area of the phytoremediation site, these measurements are within background levels of PFAS in soil measured in other studies (**Figure S6**).[8,9] PFAS contamination is widespread and global, so PFAS free soil is unlikely to be found even at sites with no known sources. No data were excluded from out study based on field blank results. Control soil (n = 3) was collected from an area of the site where no hemp was planted. There were no significant differences between spring and fall PFAS concentrations (**Figure S7**). PFOS was the highest concentration analyte, at 15 ± 7 ng/g in spring soil and 22 ± 7 ng/g in fall soil (not visualized).

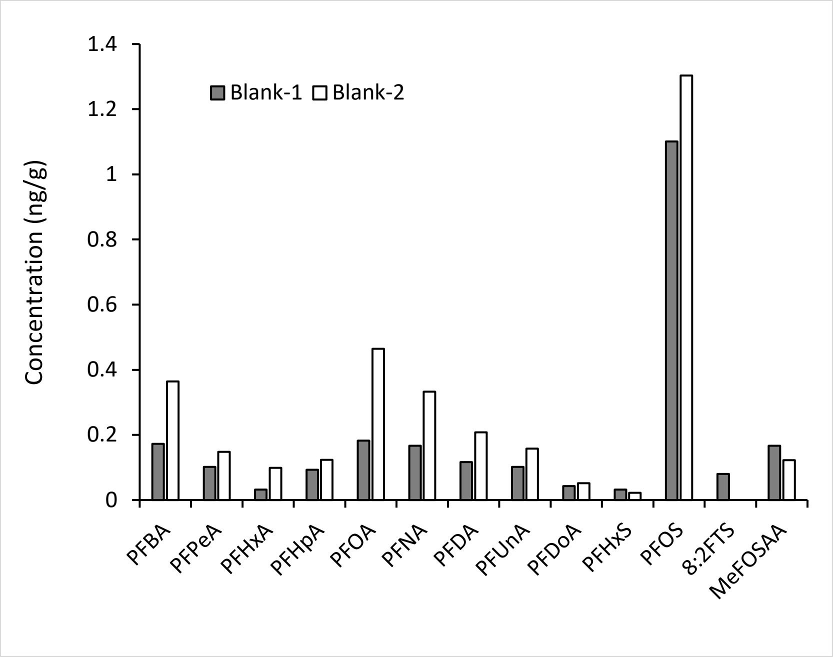

Figure S6. PFAS concentrations in field blank soil collected before (Blank-1, gray bars) and after (Blank-2, white bars) spring soil sampling, using the same equipment (n=1)

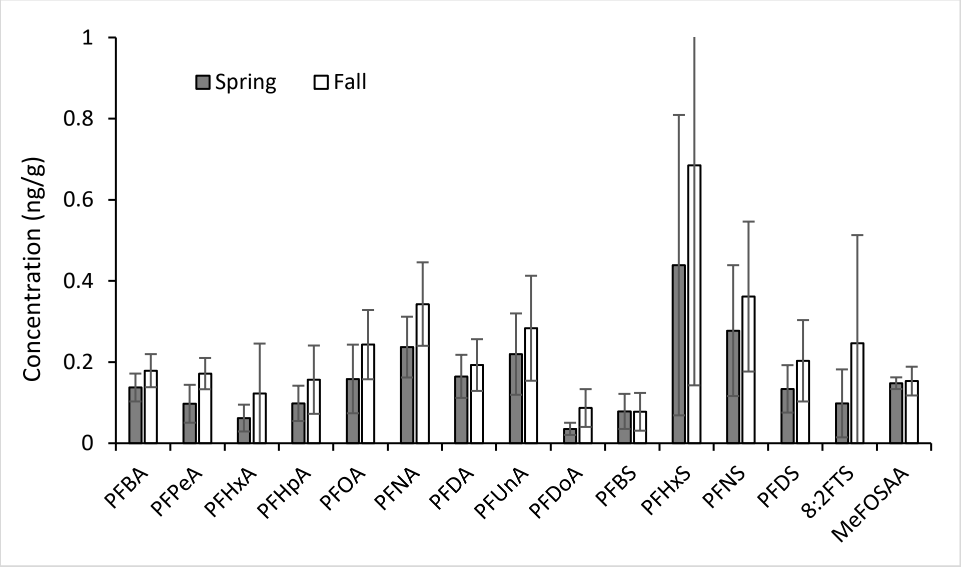

Figure S7. PFAS concentrations in control soil where no hemp was planted. Error bars show standard deviation (n = 3) There were no significant differences between fall and spring PFAS concentrations (paired t-test, $p > 0.05$).

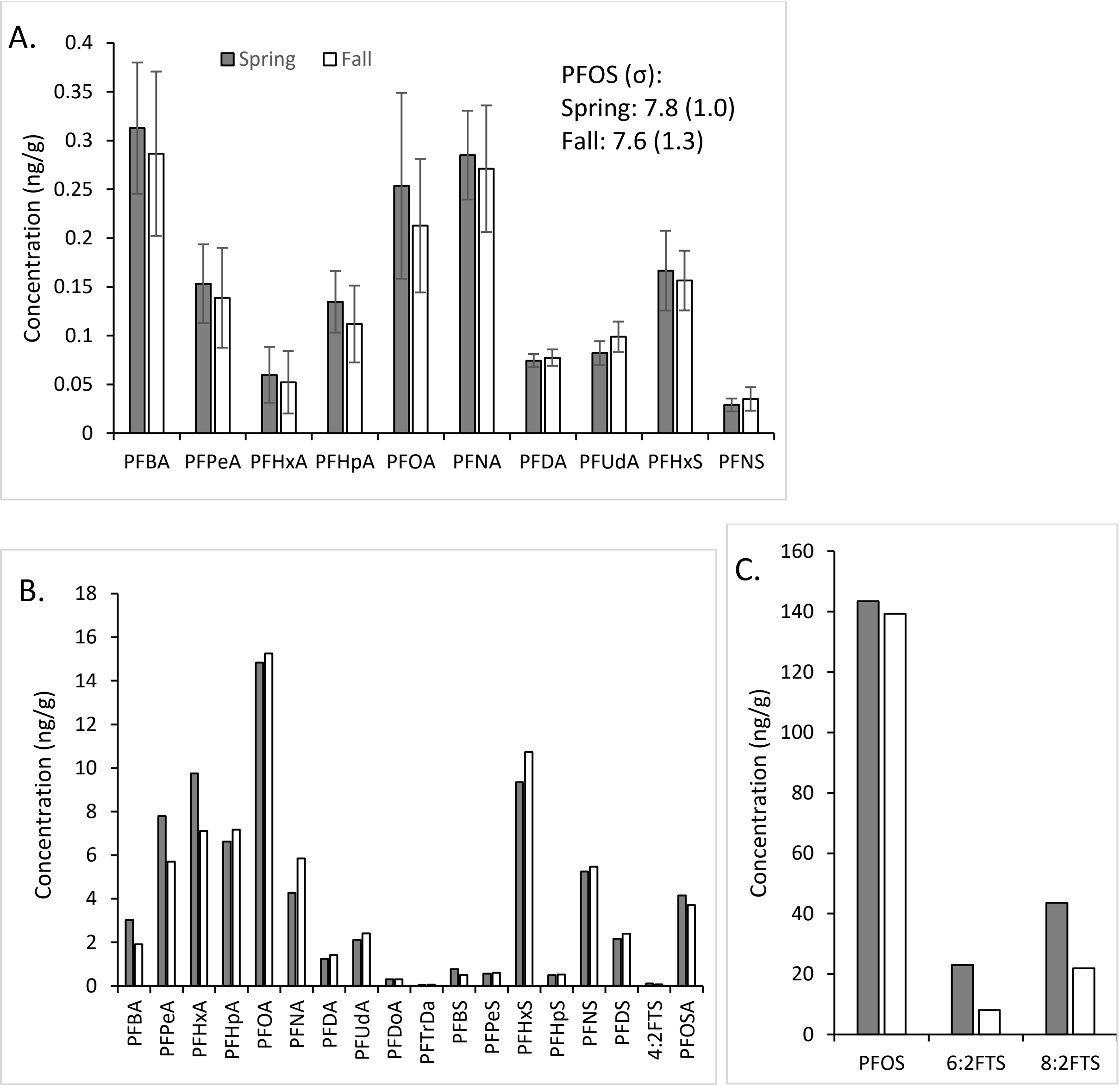

Figure S8. Comparison of spring (gray bars) and fall (white bars) soil concentrations in ChinMa hemp plots 1-4 (A) and plot 5 (B, C). Error bars in (A) show standard deviation (n=8). Statistical analysis was not possible for the plot 5 data (B, C), as there were only 2 replicates. All data were combined for comparative analysis (each PFAS tested separately, 1-tailed, paired t-tests). No significant differences were found ($p \geq 0.05$). Statistics were not performed for PFAS only detected in plot 5 (n = 2).

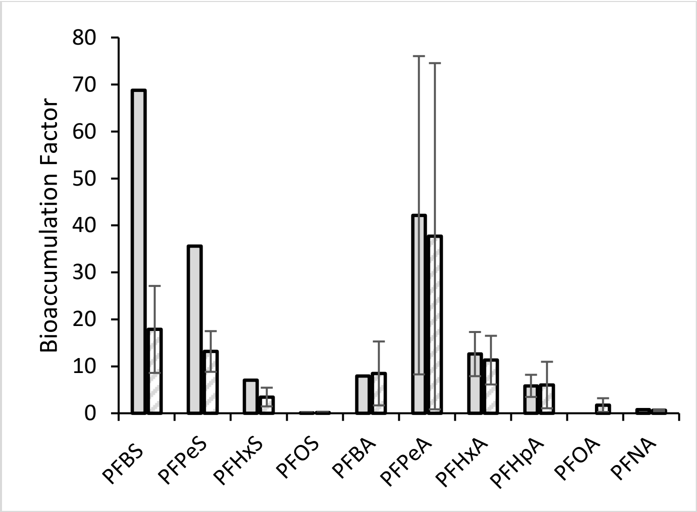

Figure S9. Comparison of leaf bioaccumulation in ChinMa hemp (gray bars) and small hemp varieties (striped bars). Error bars show standard deviation for categories where $n \geq 3$. All bioaccumulation data ($n \geq 1$ shown). Big and small hemp bioaccumulation of each PFAS was compared using t-tests when $n \geq 3$. PFOS, PFPeA, PFHxA, and PFHpA were not significantly different ($p > 0.05$) (no calculation for others).

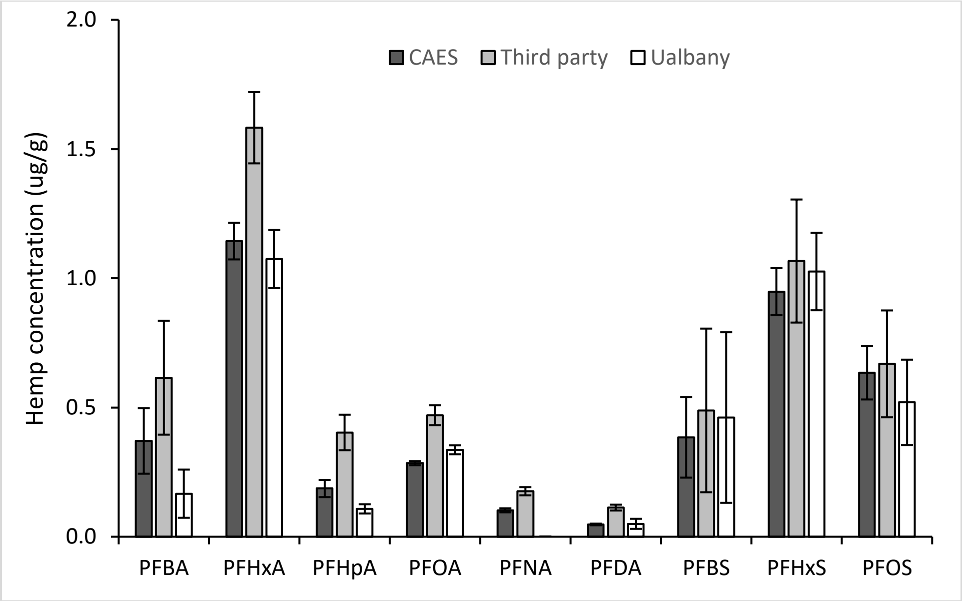

Figure S10. Comparison of results from hemp extract analysis performed by CAES, SUNY Albany, and a third party. Extractions were performed in Albany, and extracts were split and shared between labs. Error bars represent standard deviation (n=3). CAES analysis was performed using the Orbitrap HRMS method described above. A subset of HTL extracts was also analyzed at CAES to allow for investigation of NTA compounds.

References

1 S. L. Nason, J. Koelmel, N. Zuverza-Mena, C. Stanley, C. Tamez, J. A. Bowden and K. J. G. Pollitt, Software Comparison for Nontargeted Analysis of PFAS in AFFF-Contaminated Soil, *J. Am. Soc. Mass Spectrom.*, , DOI:10.1021/jasms.0c00261.

2 G. Munoz, P. Ray, S. Mejia-Avendaño, S. Vo Duy, D. Tien Do, J. Liu and S. Sauvé, Optimization of extraction methods for comprehensive profiling of perfluoroalkyl and polyfluoroalkyl substances in firefighting foam impacted soils, *Anal. Chim. Acta*, 2018, **1034**, 74–84.

3 J. P. Koelmel, P. Stelben, C. A. McDonough, D. A. Dukes, J. J. Aristizabal-Henao, S. L. Nason, Y. Li, S. Sternberg, E. Lin, M. Beckmann, A. J. Williams, J. Draper, J. P. Finch, J. K. Munk, C. Deigl, E. E. Rennie, J. A. Bowden and K. J. Godri Pollitt, FluoroMatch 2.0—making automated and comprehensive non-targeted PFAS annotation a reality, *Anal. Bioanal. Chem.*, 2022, **414**, 1201–1215.

4 W. Zhang, D. Zhang, D. V. Zagorevski and Y. Liang, Exposure of Juncus effusus to seven perfluoroalkyl acids: Uptake, accumulation and phytotoxicity, *Chemosphere*, 2019, **233**, 300–308.

5 W. Zhang and Y. Liang, Removal of eight perfluoroalkyl acids from aqueous solutions by aeration and duckweed, *Sci. Total Environ.*, 2020, **724**, 138357.

6 W. Zhang, H. Cao and Y. Liang, Plant uptake and soil fractionation of five ether-PFAS in plant-soil systems, *Sci. Total Environ.*, 2021, **771**, 144805.

7 W. Zhang and Y. Liang, Hydrothermal liquefaction of sewage sludge – effect of four reagents on relevant parameters related to biocrude and PFAS, *J. Environ. Chem. Eng.*, , DOI:10.1016/J.JECE.2021.107092.

8 Vermont Agency of Natural Resources, *Perfluoroalkyl substances (PFAS) Statewide Sampling Plan*, 2019.

9 M. L. Brusseau, R. H. Anderson and B. Guo, PFAS concentrations in soils: Background levels versus contaminated sites, *Sci. Total Environ.*, 2020, **740**, 140017.
